# Locally dose-escalated radiotherapy may improve intracranial local control and overall survival among patients with glioblastoma

**DOI:** 10.1186/s13014-018-1194-8

**Published:** 2018-12-19

**Authors:** Sebastian Zschaeck, Peter Wust, Reinhold Graf, Martin Misch, Julia Onken, Pirus Ghadjar, Harun Badakhshi, Julian Florange, Volker Budach, David Kaul

**Affiliations:** 1 Department of Radiation Oncology, Charité Universitätsmedizin Berlin, Augustenburger Platz 1, 13353 Berlin, Germany; 2 Department of Neurosurgery, Charité Universitätsmedizin Berlin, Berlin, Germany; 3 Department of Radiation Oncology, Ernst von Bergmann Medical Center, Potsdam, Germany

**Keywords:** Glioblastoma, Radiotherapy, Temozolomide, Dose escalation, Simultaneous integrated boost

## Abstract

**Background:**

The dismal overall survival (OS) prognosis of glioblastoma, even after trimodal therapy, can be attributed mainly to the frequent incidence of intracranial relapse (ICR), which tends to present as an in-field recurrence after a radiation dose of 60 Gray (Gy). In this study, molecular marker-based prognostic indices were used to compare the outcomes of radiation with a standard dose versus a moderate dose escalation.

**Methods:**

This retrospective analysis included 156 patients treated between 2009 and 2016. All patients were medically fit for postoperative chemoradiotherapy. In the dose-escalation cohort a simultaneous integrated boost of up to 66 Gy (66 Gy RT) within small high-risk volumes was applied. All other patients received daily radiation to a total dose of 60 Gy or twice daily to a total dose of 59.2 Gy (60 Gy RT).

**Results:**

A total of 133 patients received standard 60 Gy RT, while 23 received 66 Gy RT. Patients in the 66 Gy RT group were younger (*p* <  0.001), whereas concomitant temozolomide use was more frequent in the 60 Gy RT group (*p* <  0.001). Other intergroup differences in known prognostic factors were not observed. Notably, the median time to ICR was significantly prolonged in the 66 Gy RT arm versus the 60 Gy RT arm (12.2 versus 7.6 months, *p* = 0.011), and this translated to an improved OS (18.8 versus 15.3 months, *p* = 0.012). A multivariate analysis revealed a strong association of 66 Gy RT with a prolonged time to ICR (hazard ratio = 0.498, *p* = 0.01) and OS (hazard ratio = 0.451, *p* = 0.01). These differences remained significant after implementing molecular marker-based prognostic scores (ICR *p* = 0.008, OS *p* = 0.007) and propensity-scored matched pairing (ICR *p* = 0.099, OS *p* = 0.023).

**Conclusion:**

Radiation dose escalation was found to correlate with an improved time to ICR and OS in this cohort of glioblastoma patients. However, further prospective validation of these results is warranted.

**Electronic supplementary material:**

The online version of this article (10.1186/s13014-018-1194-8) contains supplementary material, which is available to authorized users.

## Background

The field of glioblastoma (GBM) treatment has seen little progress since the implementation of temozolomide (TMZ) and the establishment of trimodal therapy, which comprises surgery, adjuvant radiation with concurrent TMZ and TMZ maintenance, as standard therapy for medically fit patients by Stupp and colleagues in 2005 [1]. Tumor treating fields (TTF) during maintenance TMZ therapy have been shown to further improve patient outcome in a phase III trial compared to standard trimodal therapy [2]. However due to reimbursement issues and a potential negative impact on quality of life the addition of TTF is not yet nationwide standard of care. Currently, a standard total radiation dose of 60 Gy is administered via single-dose fractionated radiotherapy in 2-Gy fractions concomitantly with TMZ. However, this standard is based on older studies of adjuvant radiotherapy alone in which a dose-response relationship was identified for doses up to 60 Gy [3, 4].

GBM is considered a radioresistant malignancy, and most patients die from local, intracranial relapse. Therefore, radiotherapeutic dose escalation strategies have long been a topic of interest. Studies of patients treated with 2-dimensional (2D) or 3D planed radiotherapy during the pre-TMZ era reported that doses exceeding 60 Gy did not yield survival benefits; rather, patients faced an increased risk of radiation-induced brain necrosis [4, 5]. However, modern radiation techniques could potentially allow dose escalation within smaller high-risk regions with lower dose accumulations in surrounding normal brain tissues, thus decreasing the risk of symptomatic brain necrosis [6]. Several more recent studies on state-of the art dose-escalation have reported inconclusive results. One phase I study demonstrated the feasibility of dose escalation via intensity modulated radiotherapy (IMRT) without concurrent TMZ [7], while another reported promising results with a radiation dose escalation up to 81 Gy via IMRT with concomitant TMZ [8]. A phase-I/II study with a carbon ion boost suggested an association of improved progression-free survival and OS with higher carbon boost doses [9]. However, a prospective phase II study and several retrospective analyses of radiation dose escalations > 60 Gy with concomitant TMZ did not find substantial improvements in overall survival (OS) [10–12]. Accordingly, 60 Gy remains the standard radiation dose.

The conflicting findings of prior studies might be attributable to the wide variety in clinical outcome and treatment response of histologically confirmed GBM after radiotherapy and TMZ. These differences can only be partially explained by established baseline characteristics of patient, tumor and surgical resection status [13]. Accordingly, genetic alterations and molecular markers are considered important, with a high prognostic impact. Of these, O(6)-methylguanine DNA methyltransferase (MGMT) promoter methylation is likely the best known molecular marker [14]. Several other molecular parameters, such as isocitrate-dehydrogenase (IDH), have been identified. Accordingly, GBM is among the first cancers with a molecular rather than classical histological classification [15–17].

Recently, MGMT and IDH were used together with other known prognostic factors to identify subgroups of GBM patients with significantly different treatment outcomes after standard trimodal treatment [18]. The current study aimed to investigate whether a moderate state-of-the-art radiation dose escalation would correlate with an improved survival outcome in patients stratified according to these novel molecular markers.

## Methods

### Surgical treatment and radiation dose prescription

This retrospective analysis included patients treated between January 2009 and October 2016. All treatment decisions were made by an interdisciplinary tumor board.

In the standard group radiotherapy was administered in 2-Gy fractions to a total dose of 60 Gy or twice-per-day (BID) in 1.6 Gy fractions to a total dose of 59.2 Gy. These two regimens have been described in detail elsewhere and were proven to be equally efficacious in terms of OS and intracerebral failure. Patients treated with either regimen were combined in a single group for analysis (abbreviated as 60 Gy RT for ease of reading) [19].

Patients were offered an experimental regimen in which moderate dose escalation was achieved via a simultaneous integrated boost (SIB) to the macroscopic tumor region/resection cavity. This option was based on earlier in-house experiences with moderate dose escalation for GBM [5]. These patients received a 66-Gy SIB to the pre-surgical gross tumor volume (GTV) or residual disease/suspected residual tumor and 60 Gy to the surrounding planning target volume (66 Gy RT).

### Treatment planning

Computed tomography (CT) with a thermoplastic mask was performed for RT planning. Additionally, gadolinium-enhanced magnetic resonance imaging (MRI) was performed before and after surgical resection and registered rigidly to the planning CT. These MRI scans were used to delineate the target volumes and organs at risk (OAR). The GTV was defined as the combined volume of the postoperative surgical cavity, with or without residual tumor lesions, and the tumor extension on preoperative T1-weighted gadolinium-enhanced MRI. Diffusion-weighted imaging (DWI) findings were also used to estimate the GTV. The SIB volume comprised the part of the GTV identified as either residual disease or potential residual disease (e.g. eloquent areas or other critical structures that usually impede radical surgery) and was defined in collaboration with the respective neurosurgeon. Regarding the clinical target volume (CTV), a 2-cm symmetrical margin around the GTV was added with reduced margins to anatomical boundaries such as bone, tentorium or falx. For the planning target volume (PTV), an additional 0.2–0.5 cm margin was added (depending on the treatment and modality used for position verification).

IMRT was applied using a 6-MV linear accelerator with multileaf collimators or the Novalis™ therapy system (Varian, USA; Brainlab, Heimstetten, Germany). Some patients were treated using helical tomotherapy. For all patients except those undergoing tomotherapy, treatment comprised either step-and-shoot IMRT or volumetric arc therapy.

### Assessment of prognostic factors, toxicities and follow-up

According to a previous publication, patients were classified into prognostic groups (RPA [recursive partitioning analysis] class 1, 2 or 3), which were determined based on KPS, completeness of resection, IDH and MGMT status and age, as described elsewhere in detail [18]. Patient files were screened retrospectively for the analysis of toxicities, which were usually scored weekly during treatment according to the common toxicity criteria for adverse events (CTCAE) 4.0. Follow-up consisted of clinical and MRI examinations every 2–3 months. Additional amino acid positron emission tomography (PET) scans were performed if the MRI investigations were inconclusive.

### Statistical analyses and software

The patient and tumor characteristics were compared between the two treatment groups using the chi-squared test (categorical variables) or Mann–Whitney U test (continuous variables). The Kaplan–Meier method was used to calculate the OS and intracranial control (ICC) probabilities from the day of surgery, and the log-rank test was used for intergroup comparisons of these probabilities. Univariate and multivariate analyses were performed using Cox regression analyses; here, parameters with significance according to the univariate analysis (*p* <  0.05) were included in the multivariate analysis. Propensity-scored matching (PSM) was performed using the nearest-neighbor matching method with a caliper of 0.4 and matching ratio of 1:2 (dose escalation versus no dose escalation). All statistical analyses were performed using SPSS version 24.0 (IBM Inc., Armonk, NY, USA) and R version 3.2.5 (R Foundation for Statistical Computing, Vienna, Austria) [20].

## Results

Table 1 presents the baseline characteristics for both treatment groups. The patients receiving standard doses and dose escalation differed significantly in terms of age, with a median age of 51 years in the 66 Gy RT versus 62 years in the 60 Gy RT group (*p* <  0.001). In contrast, the RPA class and resection completeness did not differ between the groups. Patients in the 60 Gy RT group more frequently received concomitant TMZ (*p* <  0.001) and had a slightly larger PTV volumes.

**Table 1:** Patient and tumor characteristics

	66 Gy RT (*n* = 23)		60 Gy RT (*n* = 133)		*p*-value
Sex					
Female	12	(52.2%)	44	(33.1%)	0.172
Male	11	(47.8%)	89	(66.9%)	
Median age (years)	51		62		< 0.001
Tumor location					
Frontal	8	(34.8%)	42	(31.6%)	0.692
Parietal	5	(21.7%)	32	(24.1%)	
Temporal	4	(17.4%)	39	(29.3%)	
Occipital	2	(8.7%)	9	(6.8%)	
Central	3	(13.0%)	9	(6.8%)	
Multifocal	1	(4.3%)	2	(1.5%)	
Resection status					
Gross total	8	(34.8%)	54	(40.9%)	0.588
Subtotal	13	(56.5%)	60	(45.5%)	
Biopsy	2	(8.7%)	18	(13.6%)	
RPA class					
1	2	(8.7%)	14	(10.5%)	0.682
2	12	(52.2%)	74	(55.6%)	
3	3	(13.0%)	8	(6.0%)	
n.a.	6	(26.1%)	37	(27.8%)	
MGMT					
Methylated	11	(47.8%)	43	(32.3%)	0.163
Unmethylated	6	(26.1%)	63	(47.4%)	
n.a.	6	(26.1%)	27	(20.3%)	
PTV ml (Average)	293		357		< 0.001
Concurrent temozolomide					
Yes	18	(78.3%)	129	(97.0%)	< 0.001
No	5	(21.7%)	4	(3.0%)	

*RT* radiotherapy, *RPA* Recursive partitioning analysis, *n.a*. not available, *MGMT* O(6)-methylguanine DNA methyltransferase, *PTV* planning target volume

In the experimental patient cohort, the median SIB volume was 19.2 cm^3^ (ccm, range: 2.6–83.6 cm^3^). Dose escalation within a small volume was generally well tolerated, as indicated by a lack of differences in acute toxicity between the 60 Gy RT and 66 Gy RT groups. Additional file 1: Table S1 summarizes the observed acute toxicities within the 66 Gy RT group. Although none of these patients developed symptomatic radiation-induced brain necrosis, follow-up MRI revealed asymptomatic brain necrosis in one patient, who was referred for a histological exclusion of relapse. The biopsy confirmed radionecrosis with no signs of tumor recurrence.

**Fig. 1:**
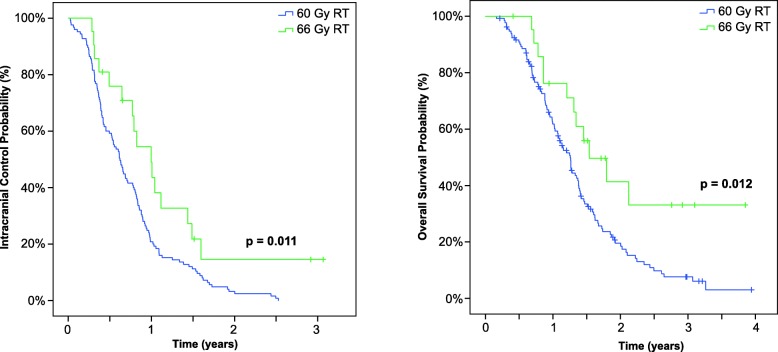
Probabilities of intracranial control (left) and overall survival among patients receiving a standard radiation dose (60 Gy radiotherapy [RT]) and those receiving dose-escalated radiotherapy (66 Gy RT). Median time to intracranial relapse: 225 days (60 Gy) versus 289 days (66 Gy), *p* = 0.011. Median OS: 397 days (60 Gy) versus 533 days (66 Gy), *p* = 0.012

**Fig. 2:**
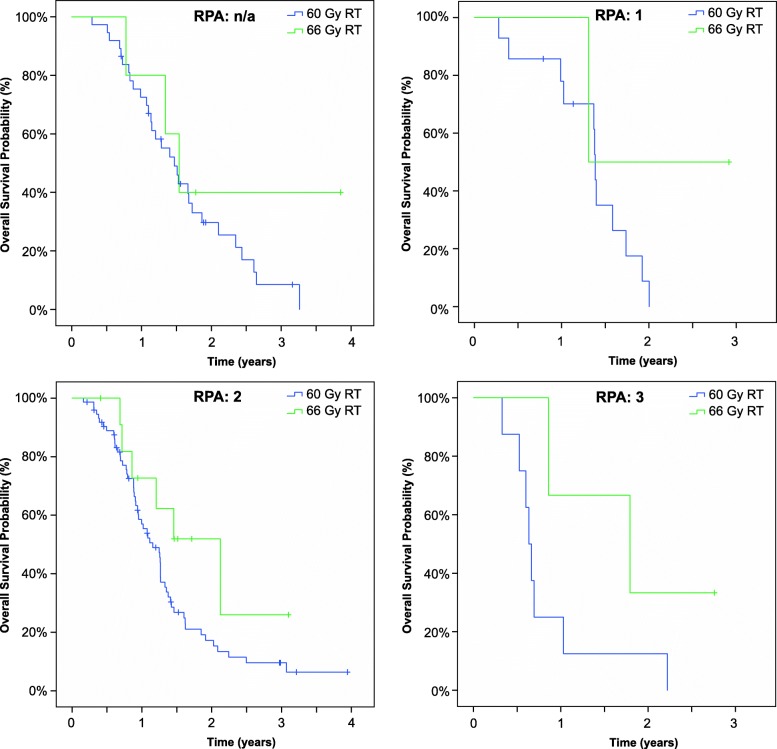
Overall survival probabilities of patients receiving a standard radiation dose (60 Gy radiotherapy [RT]) and those receiving dose-escalated radiotherapy (66 Gy RT) after stratification by recursive partitioning analysis (RPA) prognostic groups 1–3, n.a. = insufficient data for allocation. *p* = 0.007 for OS and *p* = 0.008 for ICC pooled according to RPA classification

Regarding survival, the median OS durations were 15.3 months (range 2 to 48.1 months) in the 60 Gy RT group and 18.8 months (range: 5 to 37.8 months) in the 66 Gy RT group, and the median intervals to intracranial relapse were 7.6 (range: 0.3 to 30.8 months) and 12.2 (range: 3.5 to 37.4) months, respectively. Both differences were significant, as shown in Fig. 1. To rule out confounding errors an additional comparison of the experimental cohort with the normofractionated cohort was performed. This analysis also confirmed the significant benefit regarding OS (*p* = 0.009) and intracranial relapse (*p* = 0.015) for patients treated with 66 Gy RT (Additional file 2: Figure S1). The RPA classification revealed a non-significant difference according to RPA. Additional file 3: Figure S2 depicts the corresponding Kaplan–Meier survival curves for the whole cohort. An additional analysis stratified by RPA group revealed increases in the durations of OS and ICC in the 66 Gy RT group relative to other groups (*p* = 0.007 for OS and *p* = 0.008 for ICC). Figure 2 and Additional file 4: Figure S3 present the Kaplan–Meier curves for OS and ICC by RPA class.

In the univariate analysis of factors related to OS, dose escalation correlated with an increase in OS [hazard ratio (HR) = 0.47, range: 0.26–0.86, p = 0.007], whereas a higher age (HR = 1.02, range: 1.001–1.04, *p* = 0.03) and worse resection status (HR = 1.36, range: 1.02–1.80, *p* = 0.04) were associated with a decreased OS. Furthermore, an inverse trend was observed between the RPA class and OS (HR = 1.18, range: 0.98–1.42, *p* = 0.07). Dose escalation (*p* = 0.023) and resection status (*p* = 0.028) remained significantly associated with OS in the multivariate analysis. A further univariate analysis of factors affecting ICC revealed strong associations with an escalated radiation dose (HR = 0.51, range: 0.30–0.87, *p* = 0.007) and resection status (HR = 1.35, range: 1.03–1.77, *p* = 0.031). Both factors remained significantly associated with ICC in the multivariate analysis (*p* = 0.010 and 0.021, respectively). Table 2 summarizes the results of the univariate and multivariate analyses.

**Table 2:** Univariate and multivariate Cox regression analyses of OS and ICC

	Univariate Analysis		Multivariate Analysis	
	HR (range)	*P*-value	HR (range)	*P*-value
OS				
Age	**1.020 (1.001–1.039)**	**0.034**	1.015 (0.996–1.035)	0.121
Dose escalation	**0.472 (0.259–0.858)**	**0.014**	**0.494 (0.269–0.906)**	**0.023**
Localization	1.064 (0.924–1.225)	0.387		
Resection status	**1.338 (1.009–1.772)**	**0.043**	**1.369 (1.035–1.811)**	**0.028**
No Temozolomide	1.421 (0.624–3.232)	0.403		
RPA Class	1.183 (0.982–1.424)	0.077		
PTV (ml)	1.000 (0.999–1.001)	0.542		
ICC				
Age	1.007 (0.991–1.023)	0.423		
Dose escalation	**0.512 (0.302–0.866)**	**0.013**	**0.498 (0.294–0.844)**	**0.010**
Localization	1.124 (0.976–1.295)	0.105		
Resection status	**1.344 (1.032–1.750)**	**0.028**	**1.365 (1.047–1.780)**	**0.021**
No Temozolomide	1.857 (0.866–3.983)	0.112		
RPA	1.062 (0.891–1.267)	0.502		
PTV (ml)	1.000 (1.000–1.001)	0.304		

*OS* overall survival, *ICC* intracranial control, *HR* hazard ratio, *RPA* recursive partitioning analysis; resection status was classified as gross total resection, partial resection or biopsy

**Fig. 3:**
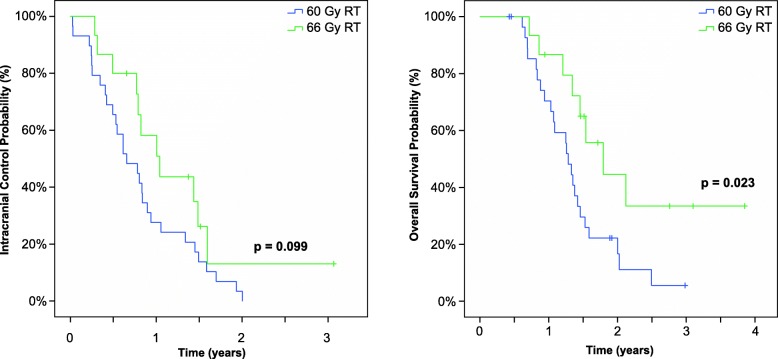
Probabilities of intracranial control (left) and overall survival patients receiving a standard radiation dose (60 Gy radiotherapy [RT]) and those receiving dose-escalated radiotherapy (66 Gy RT) after propensity-scored matching. Median OS: 457 days (60 Gy) versus 535 days (66 Gy), *p* = 0.023. Median time to intracranial relapse: 225 days (60 Gy) versus 301 days (66 Gy), *p* = 0.09

To exclude bias associated with the retrospective study design, 142 patients with fully available data were subjected to an additional PSM analysis of the following matching parameters: age, tumor location, resection type, TMZ use and RPA class (which additionally implemented information about the KPS and IDH and MGMT statuses). Here, the PSM revealed a significant difference in OS between the 66 Gy RT and 60 Gy RT groups (*p* = 0.023), while the 66 Gy RT group exhibited a trend towards better ICC (*p* = 0.099). Figure 3 presents the Kaplan-Meier plots for the propensity-matched patients. Additional file 5: Table S2 presents patient and tumor characteristics after propensity scored matching.

## Discussion

This report describes the initial experiences with state-of-the-art radiation dose escalation via a SIB approach in GBM patients stratified by molecular prognostic markers. Although the study was limited by its retrospective design, we attempted to reduce the associated bias through stratification according to molecularly defined prognostic groups and a PSM analysis, which both confirmed the initial finding that moderate dose escalation within small high-risk volumes yielded significant improvements in OS and ICC. Furthermore, this survival benefit was maintained when patients were grouped according to established prognostic groups based mainly upon the MGMT and IDH status. Nonetheless even RPA classification might not be able to completely rule out misbalances between the two treatment groups, especially as patients in the 66 Gy RT group showed a tendency towards a higher rate of MGMT promoter methylation. Additionally other potentially prognostic factors such as the exact anatomical tumor location [21–23] microRNA profile [24, 25], neutrophil to lymphocyte ratio [26] and functional imaging parameters [27] were not considered for risk stratification. We additionally did not assess other potential confounders such as the postoperative waiting period, although a large retrospective analysis of GBM patients revealed that this factor had no impact on patient survival [28]. Furthermore, we did not analyze the concomitant use of corticosteroid therapy, which may have a detrimental effect on OS [29]. Still, we note that this potential association remains controversial and is not proven by prospective data.

Although the groups in our study differed slightly with respect to age, the observed improvements in ICC and OS were not likely attributable to this difference.

Patients receiving standard-dose radiation had a median OS of 15.3 months, which was consistent with the OS reported by Stupp for the chemoradiotherapy arm (14.6 months) [1]. Furthermore, although patients in the 66 Gy RT arm were younger, concomitant TMZ use was less frequent in this group, mainly because of comorbidities or a poorer KPS. We further note that the radiation techniques did not differ between the groups: all patients received IMRT, which is potentially superior to 3D conformal radiotherapy [30]. Another difference is the PTV which was slightly larger in the 60 Gy RT group. This could mean that patients within the 66 Gy RT group presented smaller tumors, which is a potential confounder. However all but two patients in the 66 Gy RT were treated with high precision radiation therapy (Novalis) with reduced CTV-PTV margins (2 mm instead of 5 mm). Therefore the small (absolute) difference of PTVs might most likely be due to these circumstances and was therefore not confounding the results. This is supported by univariate analyses on OS and ICC that did not show an association with PTV volume.

Although GBM cells, and particularly GBM stem cells, are generally considered radioresistant [31], a dose-response relationship has been identified for these tumors [32, 33]. Therefore, efforts to improve therapeutic outcomes should include radiation dose escalation, among other strategies. The Radiation Therapy Oncology Group (RTOG) study 83–02, which was conducted during the pre-TMZ era, identified a dose-dependency among GBM patients receiving a hyperfractionated accelerated dose-escalated radiation schedule [34]. In contrast, the randomized RTOG 93–05 trial did not observe an improved outcome after dose escalation by a stereotactic boost [35].

In the TMZ era, several studies on the effect of radiation dose escalation have yielded conflicting results. One single-arm study reported a median OS of 14.8 months after a dose escalation to 72 Gy with an amino acid PET-based integrated boost. However, that study included only 22 patients, and therefore the results should be interpreted cautiously [10]. In a retrospective analysis, no significant survival benefit was observed among 128 patients receiving dose-escalated radiotherapy when compared to 81 patients receiving a standard radiation dose. In that analysis, however, the prescribed doses within the escalated arm differed widely (61–76 Gy), and the median 2-Gy equivalent dose of 64 Gy was relatively low [12]. Another retrospective analysis of 49 patients receiving dose escalations of up to 70 Gy also failed to report a significant survival benefit relative to a 60-Gy radiation dose [11]. In that analysis, however, the 60-Gy cohort had a relatively long OS of 18.4 months. Additionally, a statistical trend toward improved survival was observed for patients receiving an escalated dose to the subventricular zones was found, suggesting that the area of dose escalation is a critical factor in terms of efficacy. Several other publications highlighted that the dose to the subventricular zones may be an important factor in terms of outcome [36, 37]. This was not assessed in our study and is a potential confounder.

A study on upfront dose escalation by 6 to 14 Gy Gamma Knife radiotherapy reported very promising OS results with a median OS time of 23 months in a relatively large cohort of 174 patients [38].

The use of amino acid PET to determine target volumes for dose escalation appears to be promising. A recent study of a relatively high PET-based dose escalation reported a median OS of 20.0 months [39], and Tsien and colleagues reported a similarly promising survival outcome after dose escalation (20.1 months) [8]. The neurooncologic community eagerly awaits the results of larger ongoing studies investigating the association of amino acid PET tracer uptake with recurrence patterns (https://clinicaltrials.gov/ct2/show/NCT01873469?term=NCT01873469&rank=1), as these are expected to direct future studies of image-guided dose escalation. According to several studies, amino-acid PET-based treatment planning yields larger high-risk volumes [40] that correlate more strongly with recurrent disease than MRI-based delineation [8, 41–43]. Given the prognostic relevance of the metabolic active tumor volume [27, 44], the selective dose escalation of PET-defined volumes appears promising.

## Conclusion

In conclusion, our data suggest an association of modern approaches to radiation dose escalation within small high-risk regions with improved survival in GBM patients. However, these findings should be interpreted cautiously, given the retrospective nature of the data. Further validation through prospective trials, ideally combined with functional imaging, is warranted.

## Additional files

Additional file 1: **Table S1.** Radiation induced side effects 66 Gy RT. (DOCX 15 kb)

Additional file 2: **Figure S1.** Intracranial control probability and overall survival probability according to the three different radiation schedules: 2 Gy daily up to 60 Gy (60 Gy), 60 Gy with 66 Gy simultaneous integrated bosst (66 Gy) and bi-daily 1.6 Gy to 59.2 Gy (59.2 Gy BID). *P*-values are given for comparison between 60 Gy and 66 Gy. (PDF 36 kb)

Additional file 3: **Figure S2.** Intracranial control probability and overall survival probability according to RPA classification for all patients. (PDF 32 kb)

Additional file 4: **Figure S3.** Intracranial control probability according to RPA classification separated for patients treated with standard dose (60Gy) or with dose escalation by simultaneous integrated boost (66 Gy). (PDF 37 kb)

Additional file 5: **Table S2.** Patient and tumor characteristics after propensity scored matching. (DOCX 22 kb)

## Abbreviations

2D: 2-dimensional
BID: Twice-per-day
ccm: Cubic centimeters
CT: Computed tomography
CTV: Clinical target volume
DWI: Diffusion-weighted imaging
GBM: Glioblastoma
GTV: Gross tumor volume
Gy: Gray
HR: Hazard ratio
ICC: Intracranial control
ICR: Intracranial relapse
IDH: Isocitrate-dehydrogenase
IMRT: Intensity modulated radiotherapy
MGMT: O(6)-methylguanine DNA methyltransferase
MRI: Magnetic resonance imaging
OAR: Organs at risk
OS: Overall survival
PET: Positron emission tomography
PSM: Propensity-scored matching
PTV: Planning target volume
RPA: Recursive partitioning analysis
SIB: Simultaneous integrated boost
TMZ: Temozolomide
