# Supplementary material for: Locally dose-escalated radiotherapy may improve intracranial local control and overall survival among patients with glioblastoma
Source: Radiat Oncol. 2018 Dec 19;13:251. doi: 10.1186/s13014-018-1194-8 (PMC6299982; doi:10.1186/s13014-018-1194-8)
Supplement: Supplementary file 1 — Table S1. Radiation induced side effects 66 Gy RT. (DOCX 15 kb) [file 13014_2018_1194_MOESM1_ESM.docx]

**Table S1: Radiation induced side effects 66 Gy RT**

| **Fatigue** |  |
| --- | --- |
| 0 | 17 |
| 1 | 5 |
| 2 | 1 |
| **Dermatitis** |  |
| 0 | 14 |
| 1 | 7 |
| 2 | 2 |
| **Alopecia** |  |
| 0 | 0 |
| 1 | 23 |
| **Cognitive decline** |  |
| 0 | 21 |
| 1 | 2 |
| **Headache** |  |
| 0 | 21 |
| 1 | 2 |
