# Supplementary figures and images for: Locally dose-escalated radiotherapy may improve intracranial local control and overall survival among patients with glioblastoma
Source: Radiat Oncol. 2018 Dec 19;13:251. doi: 10.1186/s13014-018-1194-8 (PMC6299982; doi:10.1186/s13014-018-1194-8)

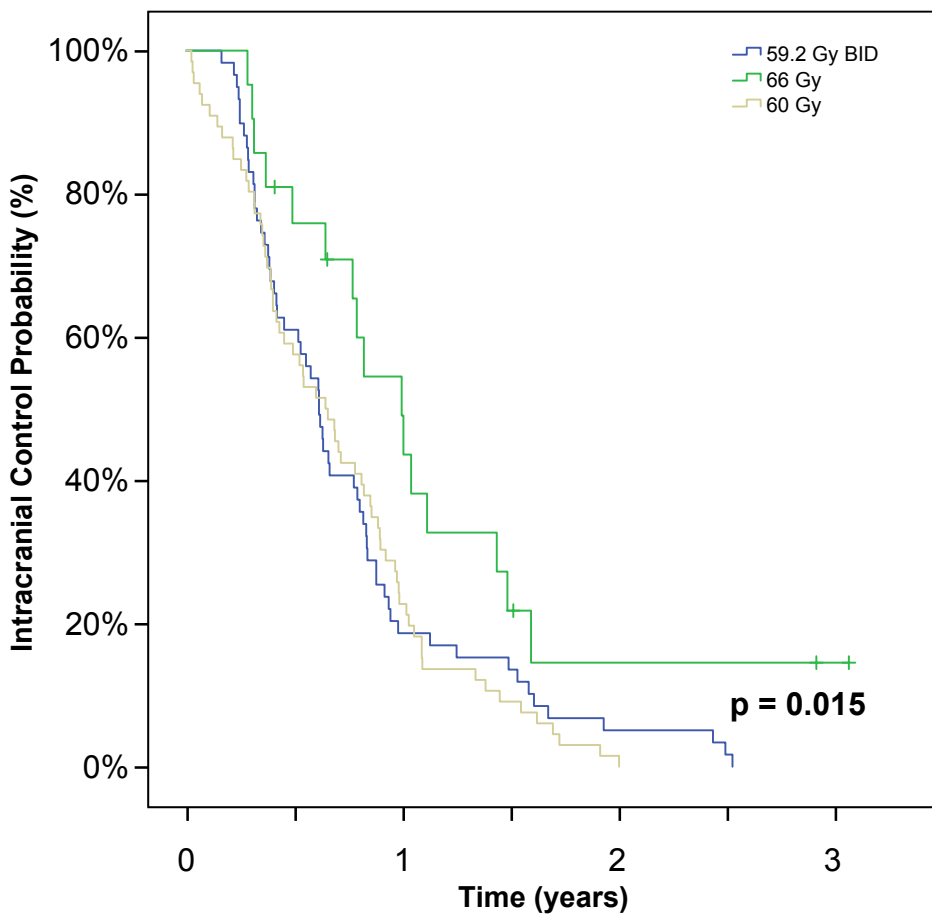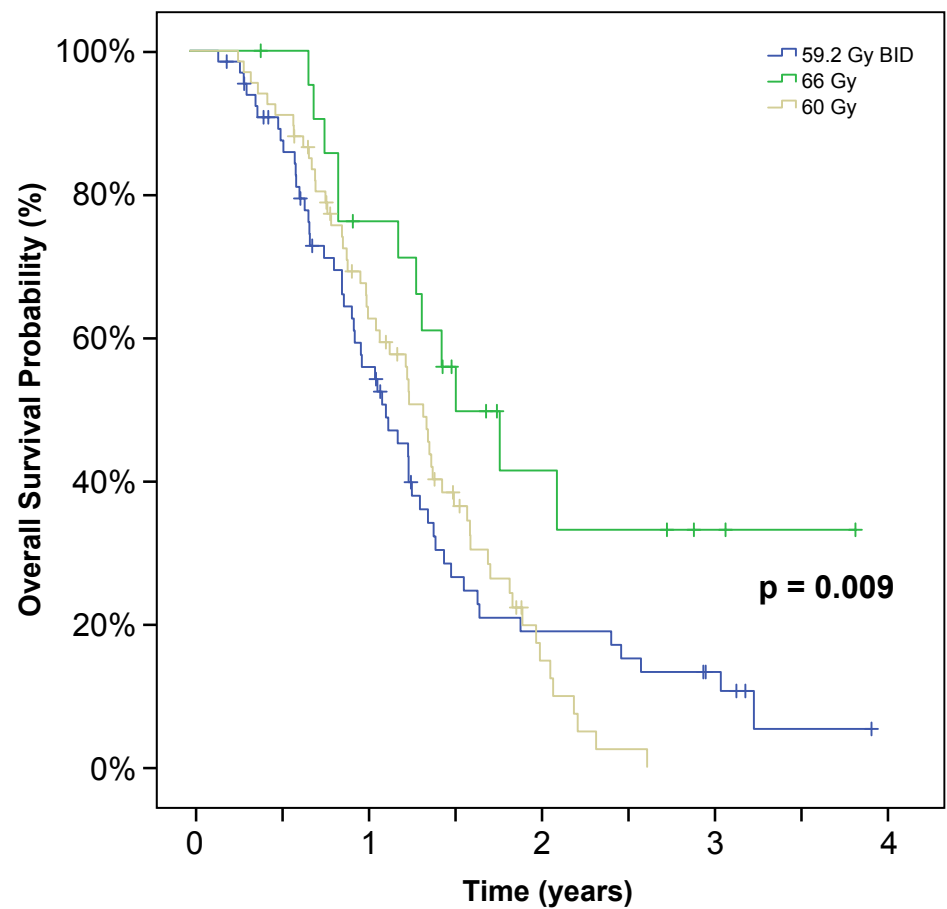

Supplement: Supplementary file 2 — Figure S1. Intracranial control probability and overall survival probability according to the three different radiation schedules: 2 Gy daily up to 60 Gy (60 Gy), 60 Gy with 66 Gy simultaneous integrated bosst (66 Gy) and bi-daily 1.6 Gy to 59.2 Gy (59.2 Gy BID). P-values are given for comparison between 60 Gy and 66 Gy. (PDF 36 kb) [file 13014_2018_1194_MOESM2_ESM.pdf]

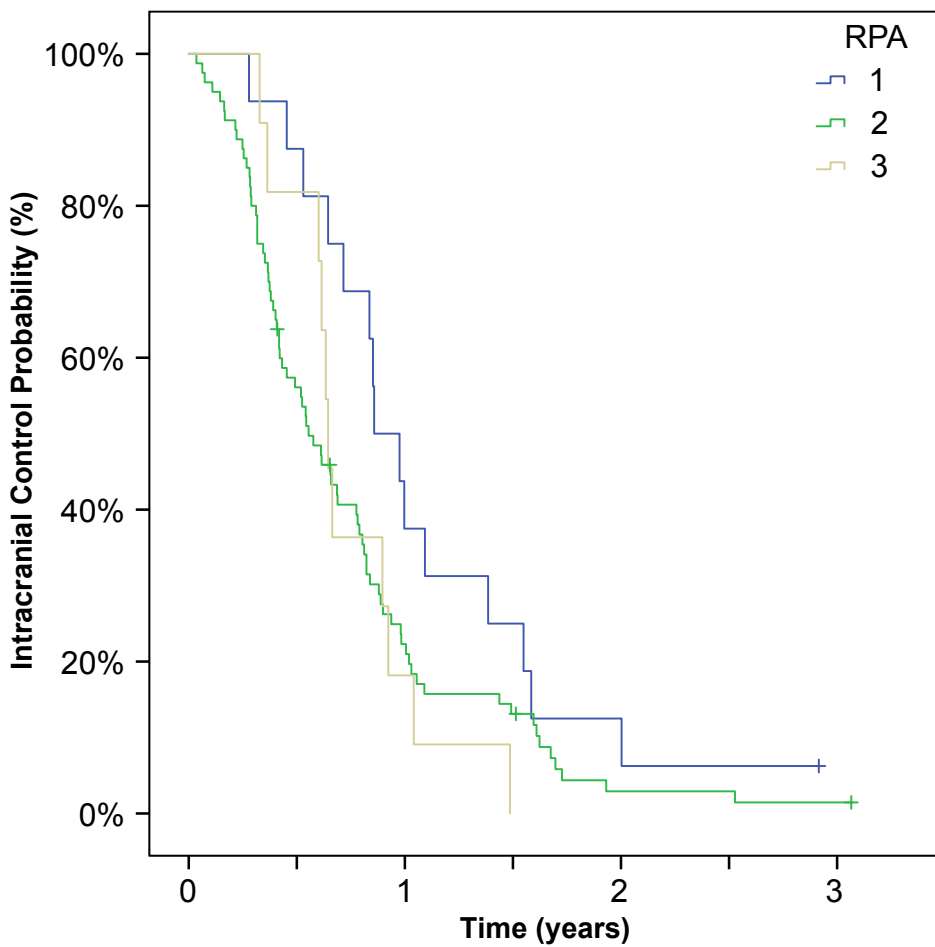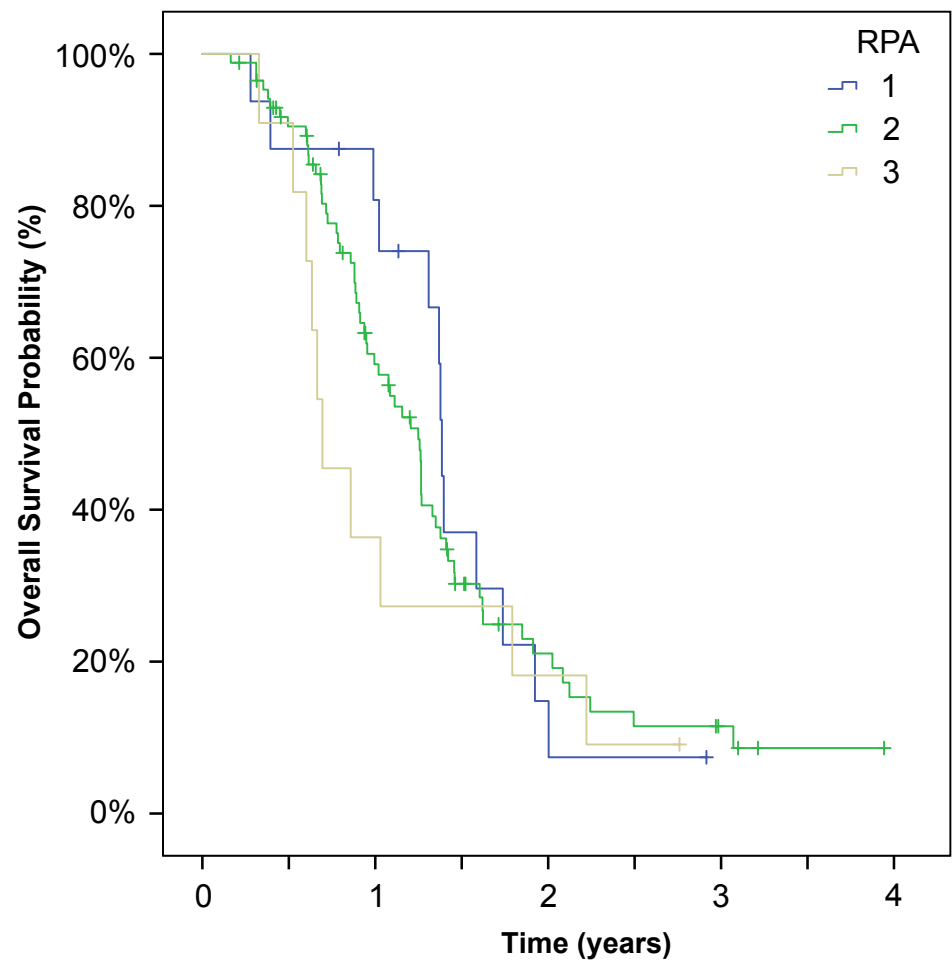

Supplement: Supplementary file 3 — Figure S2. Intracranial control probability and overall survival probability according to RPA classification for all patients. (PDF 32 kb) [file 13014_2018_1194_MOESM3_ESM.pdf]

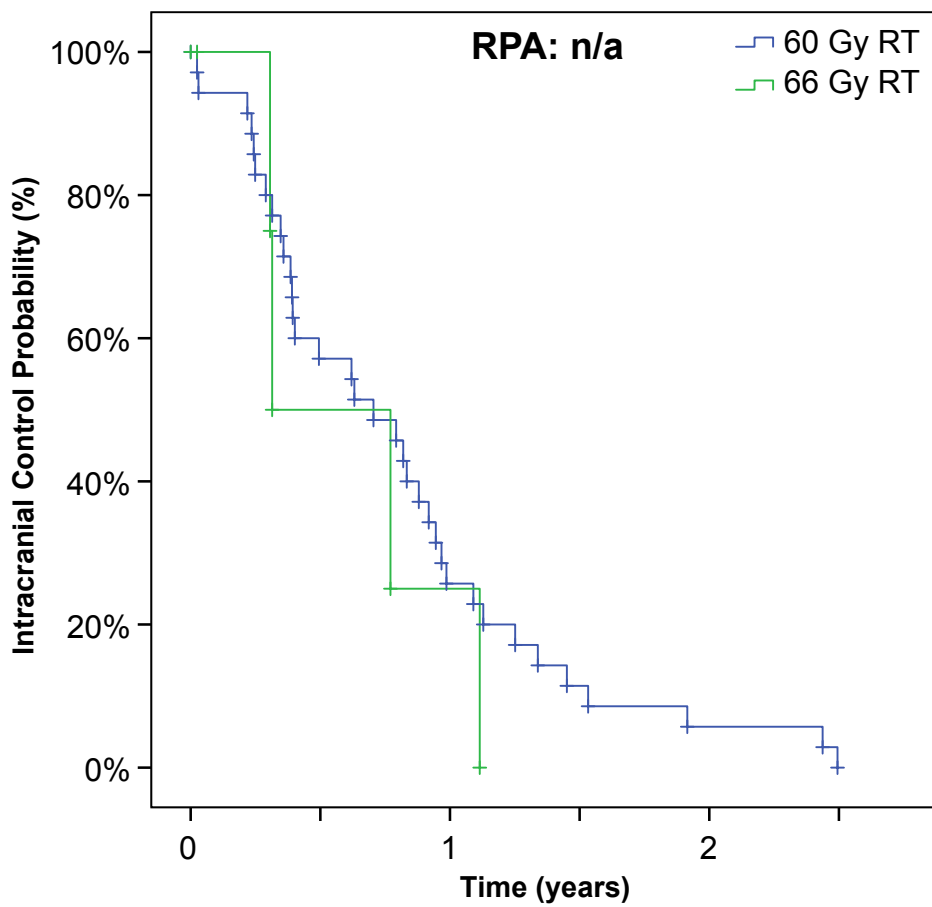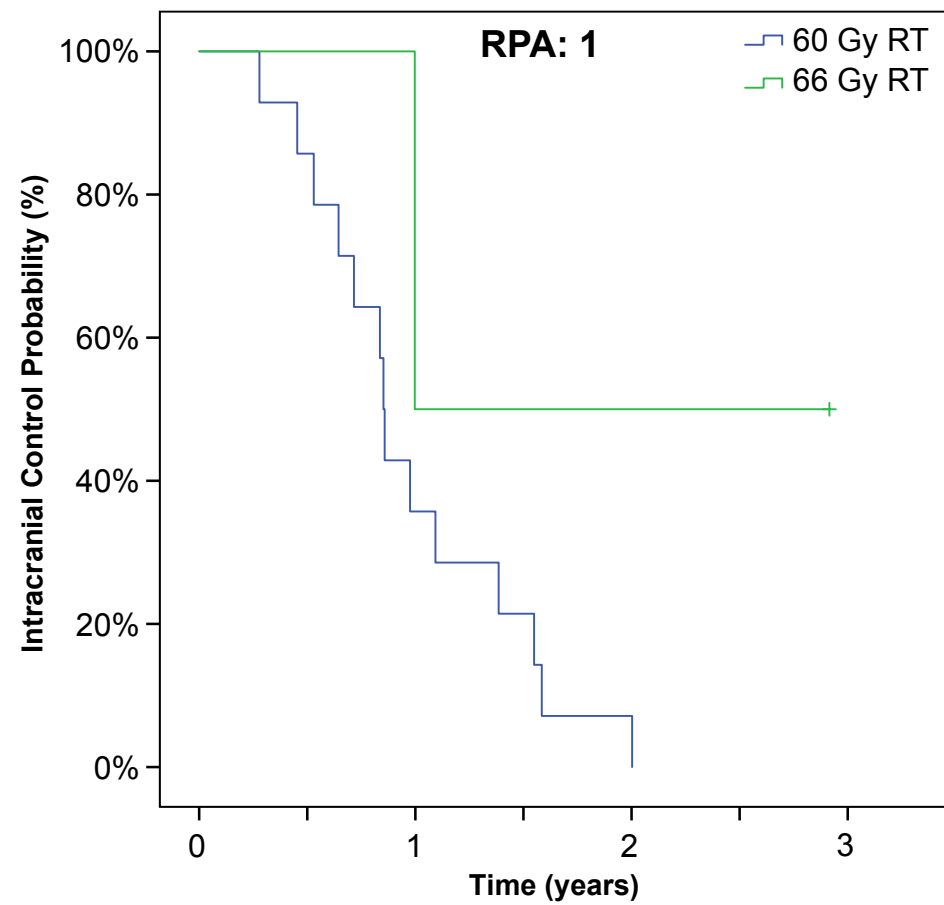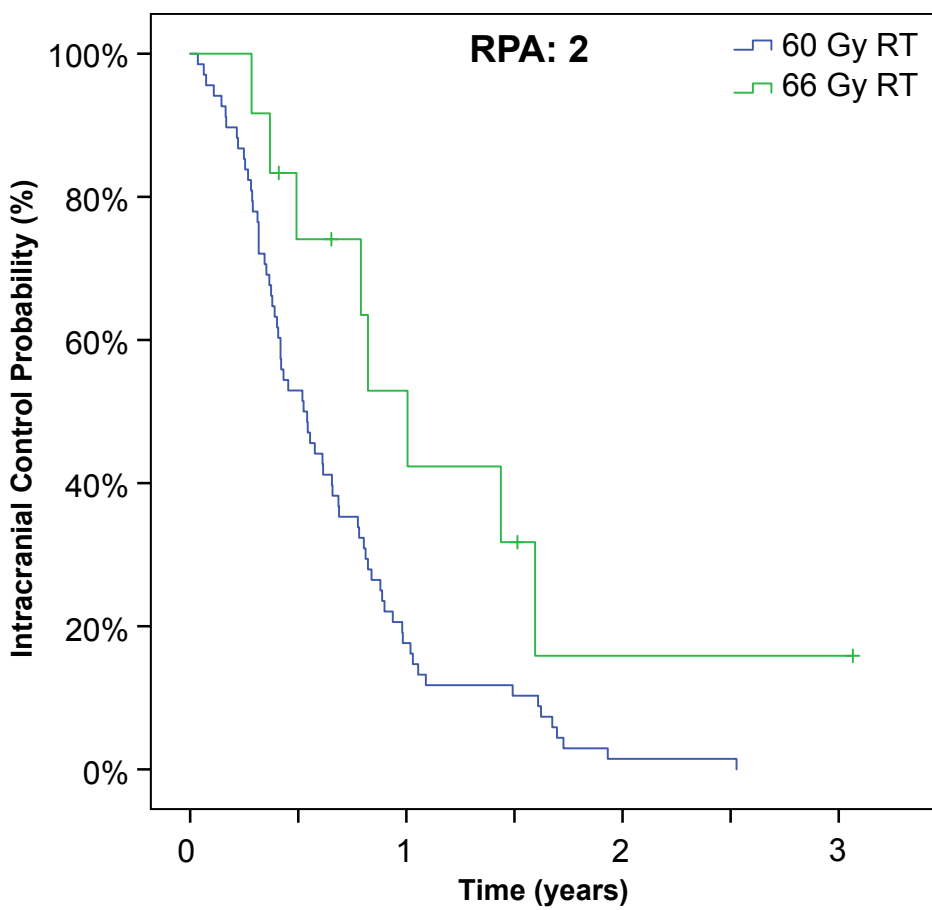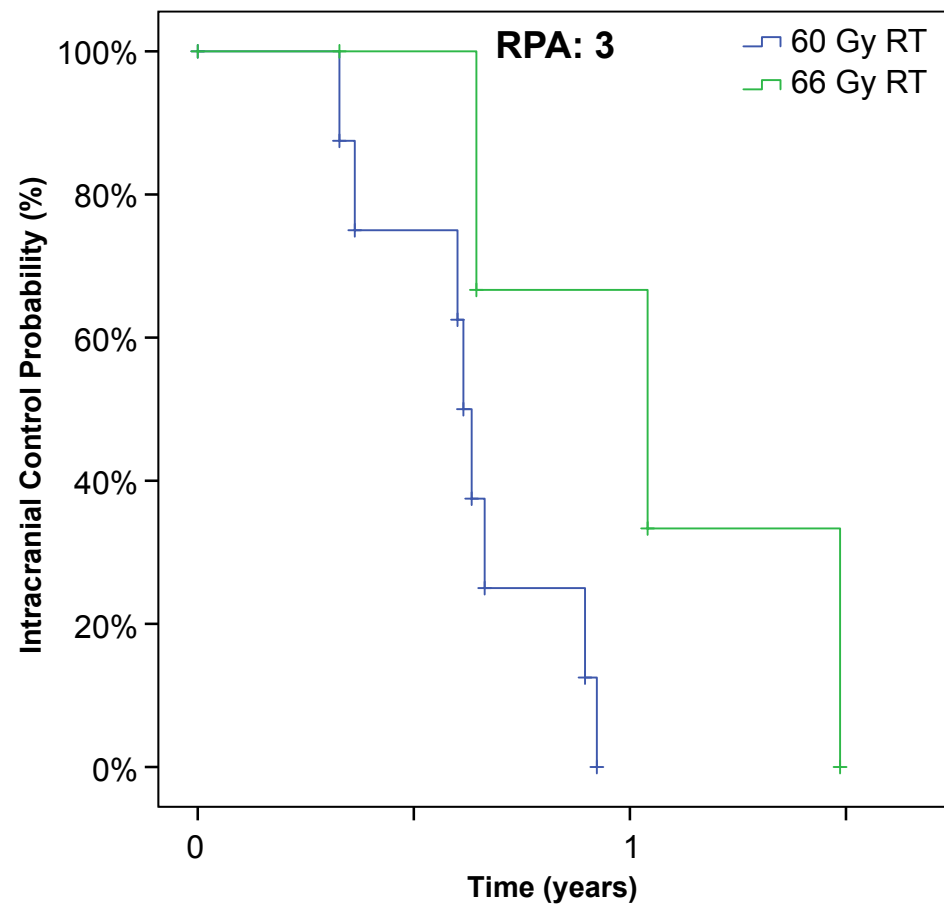

Supplement: Supplementary file 4 — Figure S3. Intracranial control probability according to RPA classification separated for patients treated with standard dose (60Gy) or with dose escalation by simultaneous integrated boost (66 Gy). (PDF 37 kb) [file 13014_2018_1194_MOESM4_ESM.pdf]
