# Supplementary material for: Locally dose-escalated radiotherapy may improve intracranial local control and overall survival among patients with glioblastoma
Source: Radiat Oncol. 2018 Dec 19;13:251. doi: 10.1186/s13014-018-1194-8 (PMC6299982; doi:10.1186/s13014-018-1194-8)
Supplement: Supplementary file 5 — Table S2. Patient and tumor characteristics after propensity scored matching. (DOCX 22 kb) [file 13014_2018_1194_MOESM5_ESM.docx]

**Table S2: Patient and tumor characteristics after propensity scored matching**

|  | **66 Gy RT (n=15)** | | **60 Gy RT (n=27)** | |  |
| --- | --- | --- | --- | --- | --- |
| **Sex** |  |  |  |  |  |
| Female | 6 | (40%) | 10 | (37%) |  |
| Male | 9 | (60%) | 17 | (63%) |  |
| **Median age** (years) | 52 |  | 58 |  |  |
| **Tumor location** |  |  |  |  |  |
| Frontal | 4 | (27%) | 10 | (37%) |  |
| Parietal | 4 | (27%) | 6 | (22%) |  |
| Temporal | 3 | (20%) | 4 | (15%) |  |
| Occipital | 2 | (13%) | 2 | (7%) |  |
| Central | 2 | (13%) | 4 | (14%) |  |
| Multifocal | 0 | (0%) | 1 | (4%) |  |
| **Resection status** |  |  |  |  |  |
| (Near) complete | 6 | (40%) | 7 | (26%) |  |
| Residual tumor | 8 | (53%) | 18 | (67%) |  |
| Biopsy | 1 | (7%) | 2 | (7%) |  |
| **MGMT** |  |  |  |  |  |
| Methylated | 8 | (54%) | 14 | (52%) |  |
| Unmethylated | 5 | (33%) | 9 | (33%) |  |
| n.a. | 2 | (13%) | 4 | (15%) |  |
| **RPA class** |  |  |  |  |  |
| 1 | 1 | (7%) | 0 | (0%) |  |
| 2 | 10 | (67%) | 18 | (67%) |  |
| 3 | 1 | (7%) | 2 | (7%) |  |
| n.a. | 3 | (20%) | 7 | (26%) |  |
| **Concurrent temozolomide** |  |  |  |  |  |
| Yes | 14 | (93%) | 24 | (89%) |  |
| No | 1 | (7%) | 3 | (11%) |  |

RT, radiotherapy; RPA, recursive partitioning analysis; n.a., not available;
